# Autoantibodies to Apolipoprotein A-1 in Cardiovascular Diseases: Current Perspectives

**DOI:** 10.1155/2012/868251

**Published:** 2012-11-20

**Authors:** P. C. Teixeira, P. Cutler, N. Vuilleumier

**Affiliations:** ^1^Translational Research Sciences, F.n Hoffman-La Roche Ltd., 4070 Basel, Switzerland; ^2^Department of Human Protein Science, Geneva Faculty of Medicine, University of Geneva, 1211 Geneva, Switzerland; ^3^Division of Laboratory Medicine, Department of Genetics and Laboratory Medicine, Geneva University Hospital Geneva, 1211 Geneva, Switzerland; ^4^Laboratory Medicine Service, Department of Genetics and Laboratory Medicine, Geneva University Hospital, 4 Rue Gabrielle-Perret-Gentil, 1211 Geneva, Switzerland

## Abstract

Immune-mediated inflammation plays a major role in atherosclerosis and atherothrombosis, two essential features for cardiovascular disease (CVD) development, currently considered as the leading cause of death in the western world. There is accumulating evidence showing that humoral autoimmunity might play an important role in CVD and that some autoantibodies could represent emerging cardiovascular risk factors. Recent studies demonstrate that IgG autoantibodies against apolipoprotein A-1 (apoA-1) are raised in many diseases associated with a high cardiovascular risk, such as systemic lupus erythematosus, acute coronary syndrome, rheumatoid arthritis, severe carotid stenosis, and end-stage renal disease. In this work, we aimed at reviewing current data in the literature pointing to anti-apolipoprotein A-1 antibodies (anti-apoA-1 IgG) as a possible prognostic and diagnostic biomarker of cardiovascular risk and appraising their potential role as active mediators of atherogenesis.

## 1. Introduction 

Immune-mediated inflammation plays a major role in atherosclerosis and atherothrombosis, two essential features for cardiovascular disease (CVD) development, currently considered as the leading cause of death in the Western world [1]. Although initially related to a lipid metabolism abnormality, atherosclerosis is now considered as a chronic multifactorial immune-mediated inflammation of the arterial wall, where transendothelial migration of circulating immuno-competent cells within the artery is the key step [1]. By fulfilling the “Koch's postulates”, recent work suggested that atherosclerotic low-grade inflammation might be even considered as an autoimmune disease [2]. This hypothesis is supported by two main pieces of evidence. Firstly, patients suffering from an autoimmune disease, such as systemic lupus erythematosus (SLE), antiphospholipid syndrome (APS), and rheumatoid arthritis (RA), display an increased cardiovascular (CV) risk, independently of the Framingham risk factors [3–5]. Secondly, in patients with overt CVD, but without concomitant autoimmune diseases, many different autoantibodies have been shown to predict poor CV outcome [6]. Some of these autoantibodies might directly influence atherosclerotic processes triggering innate immune receptors signaling either toward a pro- or an anti-inflammatory response, as reviewed elsewhere [6]. Lately, humoral autoimmunity to apolipoprotein A1 (apoA-1)—the major protein fraction of high-density lipoproteins (HDL), conferring to the latter most of its atheroprotective role [7–9]—has gained considerable interest, mostly by displaying intriguing CV prognostic and diagnostic properties in different diseases. The aim of this manuscript is to review the existing data in the literature pointing to anti-apolipoprotein A-1 antibodies (anti-apoA-1 IgG) as an emergent prognostic and diagnostic biomarker of cardiovascular risk in clinically overt autoimmune settings, as well as in nonautoimmune conditions, and to appraise their potential role as active mediators of atherogenesis according to available data in the literature.

## 2. Anti-apoA-1 IgG in Autoimmune Diseases

### 2.1. Anti-apoA-1 IgG in SLE and in APS

Anti-apoA-1 IgG were first identified in 1998 by Dinu and colleagues who demonstrated that high levels of those autoantibodies were found in a significant subset of SLE (32.5%) and primary APS patients (22.9%) and displayed a high affinity to nascent and mature HDL molecules [10]. Three years later in 2001, the group from Abe and colleagues characterized six different monoclonal anti-apoA-1 antibodies derived from two SLE patients [11]. Those autoantibodies showed a functional heterogeneity in their cross-reactivity, reacting preferentially with oxidized apoA-1 and with other self-antigens, such as cardiolipin (CL), single-strand DNA, and thrombin [11]. The relative selectivity of anti-apoA-1 IgG for its presupposed target was further confirmed two years later by Delgado Alves and colleagues, who demonstrated that anti-apoA-1 IgG antibodies cross-reacted with anti-HDL and with anti-cardiolipin antibodies [12]. Nevertheless, if 58% of SLE sera containing high levels of anti-HDL cross-reacted with CL, only 25% of those sera were reactive to apoA-1, suggesting that anti-apoA-1 IgG could represent a distinct and specific subclass of anti-HDL antibodies [12]. By demonstrating that anti-HDL IgG were inversely correlated with paraoxonase-1 (PON-1) activity and with the total antioxidant capacity of the corresponding sera [13], Batuca and colleagues were the first in 2003 to suggest that anti-HDL, and later anti-apoA-1 IgG [14], could be involved in atherogenesis, and more specifically to be related to the occurrence of dysfunctional HDL [14, 15], whose pathophysiological importance in atherogenesis started to be recognized [16]. Lately, the same group demonstrated that this effect was due to a decrease in PON-1 activity, leading to an increase of proinflammatory reactive oxygen species [17, 18], but the causal nature of this association is still under investigation. 

### 2.2. Anti-apoA-1 IgG in Rheumatoid Arthritis

The existence of anti-apoA-1 in RA patients was initially described by Vuilleumier and colleagues, in 2010, who demonstrated a high titre of those autoantibodies in 17% of RA patients [17]. Furthermore, in this single-center prospective study, of 133 subjects, the authors demonstrated that RA patients with high levels of anti-apoA-1 IgG had much worse cardiovascular event-free survival (median followup period of 9 years) when compared to patients tested negative for those autoantibodies (43% versus 9%, *P* = 0.001) [17]. In this study, being positive for anti-apoA-1 IgG (with anti-apoA-1 IgG values above the 97.5th centile of anti-apoA-1 IgG values obtained on 140 healthy blood donors) increased the risk of major adverse cardiovascular events (MACE, defined by the occurrence of fatal nonfatal acute coronary syndrome or stroke) by 4-fold (adjusted hazard ratio 4.2; 95% confidence interval (95% CI): 1.5–12.1). This was independent of traditional cardiovascular risk factors and RA disease duration [17]. Finally, patients tested positive for those autoantibodies were shown to have higher plasma levels of inflammatory mediators associated with atherosclerotic plaque vulnerability in humans, such as matrix metalloproteinase 9 (MMP-9) and oxidized low-density lipoproteins (oxLDL) [18, 19]. 

These results were corroborated in a smaller case-control Swedish study which confirmed the aforementioned association with oxLDLs and demonstrated that anti-apoA-1 IgG levels were higher in RA patients than in control subjects and were higher in RA patients with a history of MACE when compared to those without [20]. Finally, a recent study demonstrated that when compared to 6 different CV prognostic biomarkers, anti-apoA-1 IgG was the only one found to provide incremental predictive ability over the 10-year global Framingham risk score, improving the area under the curve from 0.72 to 0.81, and it improved reclassification statistics using the integrated discrimination index (IDI) by 175% (*P* < 0.001) [21]. Thus, in this study, anti-apoA-1 IgG was found to be the only candidate to provide better and complementary prognostic information to traditional cardiovascular risk factors.

Taken together, those results suggest that anti-apoA-1 IgG have a CV prognostic value mostly in RA patients, where they are associated with proinflammatory cytokine profile and appear to provide incremental predictive ability over Framingham risk factors. Nevertheless, whether this CV prognostic feature also applies to SLE and APS patients is currently elusive and warrants further studies. Finally, knowing how and if assessing anti-apoA-1 IgG could impact the actual CV risk stratification and subsequent therapeutic management of RA patients remains to be demonstrated.

## 3. Anti-apoA-1 IgG in Nonautoimmune Conditions

### 3.1. Anti-apoA-1 IgG in Acute Coronary Syndromes

The existence of anti-apoA-1 IgG in nonautoimmune settings was initially reported in a retrospective study published in 2004, which demonstrated that the prevalence of high levels of anti-apoA-1 IgG was higher in myocardial infarction (MI) patients than in healthy blood donors (21% versus 1%; *P* = 0.001) [22]. This prevalence was of the same order of magnitude as previously reported in SLE and APS patients [10–13], and in this work, the authors retrieved a significant association between anti-apoA-1 IgG and serum amyloid A (SAA) protein, a multifunctional protein located at the crossroad of inflammation, cholesterol homeostasis, and atherogenesis [23, 24]. The findings from this study were confirmed and extended by a case-control study from the same group involving 127 patients with acute coronary syndrome (ACS), 140 healthy blood donors, 34 patients with stroke, and 58 patients with acute pulmonary embolism [25]. In this study, the prevalence of high anti-apoA-1 IgG levels was 11% in the ACS group, 2% in the healthy blood donor group, and 0% in the acute pulmonary embolism group. In ACS patients, those considered as positive for anti-apoA-1 IgG (with anti-apoA-1 IgG values above the 97.5th centile of anti-apoA-1 IgG values obtained from 140 healthy blood donors) had significantly higher median levels of oxLDL when compared to ACS patients tested negative for those autoantibodies (*P* < 0.0001) [25]. In this study, the presence of any autoimmune disease was excluded based upon the study exclusion criteria, and all the patients tested positive for anti-apoA-1 IgG were found to be negative for anti-*beta2* glycoprotein (anti-*β*2GPI), rheumatoid factor (RF), anti-nucleosome, anti-nucleoprotein-specific, and for ANCA-specific anti-MPO (myeloperoxidase) and anti-PR3 (proteinase 3) antibodies [25].

Two years later, the same group demonstrated the prognostic value of anti-apoA-1 IgG on a single center prospective study, including 221 patients hospitalized for acute MI, who all completed a one-year followup [26]. In this study, patients which had high levels of anti-apoA-1 IgG upon admission had a worse cardiovascular event-free survival at one year than patients tested negative for those autoantibodies (63.2% versus 88.5%, *P* = 0.001). Risk analyses indicated that being positive for anti-apoA-1 antibodies increased the subsequent risk of major adverse cardiovascular events (MACE, consisting of fatal or nonfatal ACS or stroke and hospitalization for acute heart failure) by 4-fold, independently of traditional CV risk factors (OR, 4.3; 95% CI, 1.46–12.6; *P* = 0.007). Furthermore, when compared to patients with low anti-apoA-1 IgG levels, those tested positive for anti-apoA-1 IgG were found to have: (i) higher basal heart rate upon discharge, a well-established cardiovascular prognostic feature after MI [27–29], and (ii) a higher proinflammatory cytokine profile in plasma, possibly associated with atherosclerotic plaque vulnerability [30].

Because many different autoantibodies have been shown to yield significant CV prognostic information, as extensively reviewed in [6], the same investigators undertook an ancillary study on the same cohort of patients comparing in a “head-to-head” manner the prognostic accuracies of autoantibodies to *β*2GPI domains I and IV, cardiolipin, apolipoproteinA-1 (anti-apoA-1 IgG), heat-shock protein 60 (anti-HSP-60), and phosphorylcholine (anti-PC IgM) [31]. Based upon ROC curve analyses, anti-apoA-1 IgG was found to be the only autoantibody to significantly predict the occurrence of subsequent MACE, and a trend was observed for anti-cardiolipin and anti-HSP60 antibodies (*P* = 0.05 and *P* = 0.07, resp.). Although significant, the area under the curve (AUC) was rather modest (AUC: 0.65, *P* = 0.007) but was found to be identical to that from the 10-year global Framingham risk score, used to determine patient therapeutic management [32]. Finally, Cox regression analyses demonstrated that being positive for anti-apoA-1 IgG increased the 1-year MACE risk by 4-fold, independent of the 10-year global Framingham risk score (hazard ratio: 3.8, *P* = 0.002) [31]. Those preliminary results indicate that in secondary prevention patients, anti-apoA-1 IgG could well be the most promising humoral autoimmune candidate for MACE prediction in nonautoimmune settings. Larger multicentre randomized control trials are now needed to determine whether an anti-apoA-1 IgG-based CV risk stratification algorithm could reach clinical decision making. Until then, no clinical recommendations can be proposed.

Finally, a recent publication by Keller and colleagues suggests that anti-apoA-1 IgG could be of diagnostic utility in patients presenting to the emergency room for acute chest pain [33]. In this single-centre prospective study involving 138 patients, the investigators demonstrated that anti-apoA-1 IgG values assessed on the first sample available had a relatively good diagnostic accuracy for non-ST elevation myocardial infarction (NSTEMI), with an AUC of 0.75 (*P* < 0.0001) that could be increased up to 0.88 when combined with anti-PC IgM and the NSTEMI-TIMI score to generate the clinical antibody ratio (CABR) score. Also, anti-apoA-1 IgG was found to be a good predictor (AUC 0.80, *P* < 0.0001) of subsequent troponin I elevation when the first sample was tested negative, which was the secondary endpoint of this study. Risk analyses indicated that in the presence of high anti-apoA-1 IgG levels the risk of subsequent NSTEMI diagnosis was increased by 6-fold after the adjustment for NSTEMI-TIMI score (OR: 6.4, 95% CI: 1.72–24.2). At the prespecified cut-off, this test displayed interesting negative predictive values of 88% and 95% for the primary and secondary study endpoints, respectively. However, the positive predictive values were too low to be clinically meaningful [33]. One important limitation of this work is the lack of a suitable contemporary comparator, such as a sensitive cardiac troponin assay, known to overcome the current limitations of conventional troponin assays [34]. Whether anti-apoA-1 IgG or the CABR score could provide incremental diagnostic value over those highly sensitive assays is far from obvious and need to be challenged in further studies. Also, cost-effectiveness studies are required to determine the impact of anti-apoA-1 IgG or CABR score assessment on both patient management and its related costs.

### 3.2. Anti-apoA-1 IgG in Patients with Severe Carotid Stenosis

Montecucco and colleagues investigated whether anti-apoA-1 IgG antibodies could be retrieved in a prospective cohort of 102 Italian patients who underwent carotid endarterectomy for severe carotid stenosis and retrieved a prevalence of high anti-apoA-1 IgG levels of 20% [35]. The patients included were devoid of any known CV events of atrial fibrillation and did not display any concomitant inflammatory diseases [35]. Conventional histochemistry analyses were performed on carotid biopsies of every patient and revealed that those tested positive for anti-apoA-1 IgG (in the serum) were found to have higher intraplaque infiltration of macrophages (11% versus 7%, *P* = 0.04), neutrophils (10% versus 3%), a higher expression of MMP-9 (19% versus 5%, *P* < 0.0001), and a lower total collagen content (18% versus 21%, *P* = 0.002) when compared to patients tested negative for those autoantibodies [35]. Although there is currently no strict consensus about the exact definition of vulnerable plaque in humans [36], this phenotype fulfills some of the proposed characteristics of a vulnerable atherosclerotic plaque [37]. The possible causal nature of this association has been proposed by the investigators based upon relevant *in vitro* and animals experimental results described in detail later on. Those results suggest that anti-ApoA-1 IgG assessment could be an interesting option to identify carotid atherosclerotic plaque vulnerability, avoiding the use of unstandardized and resource demanding imaging modalities. 

As atherosclerotic plaque rupture is one of the major determinants of myocardial infarction and stroke occurrence, those observations should be accompanied by a MACE increase in patients tested positive for those autoantibodies. To test this hypothesis, a one-year followup extension of this study is currently ongoing and will include a total of 178 patients.

### 3.3. Anti-apoA-1 IgG in Patients under Chronic Dialysis

Chronic kidney disease (CKD) is well known to be associated with an increased CV risk and gave rise to the so-called cardio-renal syndrome [38]. The premature CVD observed in CKD and maintenance hemodialysis (MHD) patients has been attributed to the superimposition of both traditional CV and nontraditional CV risk factors [38, 39]. Among the nontraditional CV risk factors, humoral immune dysfunction could contribute to ESRD-related CVD [40]. To this respect, low levels of anti-atherogenic antibodies, such as anti-PC IgM, were shown to independently predict all cause mortality in MHD patients [41], whereas high levels of proatherogenic autoantibodies directed against oxLDL are increased in hemodialysis patients [40] and possibly related to CVD in those patients [42].

In a cross-sectional study involving 66 MHD patients, Pruijm and colleagues lately reported a prevalence of anti-apoA-1 IgG positivity of 20% [43]. Significant associations were retrieved between circulating levels of anti-ApoA-1 IgG and dialysis vintage, a major determinant of vascular calcification known to negatively impact MHD patient prognosis [44]. Whether the presence of anti-apoA-1 IgG could explain the occurrence of dysfunctional and proinflammatory HDL reported in CKD patients [45] remains elusive and devoid of any experimental and clinical evidences. Nevertheless, their existence in the MHD illustrates the potential role of humoral autoimmunity in CKD-related atherogenesis. Whether anti-apoA-1 IgG could yield some prognostic information in CKD or MHD patients is currently unknown and clearly warrants further studies.

## 4. Anti-apoA-1 IgG and Cardiovascular Risk: A Causal Relationship?

Clinically driven *in vitro* and animal studies tend to support a causal role between anti-apoA-1 IgG and CV risk through different mechanisms. 

First of all, when exposed to human monocytes-derived macrophages, anti-apoA-1 IgG were found to induce a dose-dependent production of different proinflammatory molecules, such as IL-8, MMP-9, IL-6, TNF-*α*, and MCP-1 [21, 30, 35]. In those studies, the exact mechanisms were not reported but the potential confounder of lipopolysaccharide contamination has been systematically addressed and reasonably excluded by Limulus assays. Further mechanistic insights were brought by Pagano and colleagues who demonstrated that the direct anti-apoA-1 IgG proinflammatory effect was mediated by their interaction with the toll-like receptor (TLR)-2/CD14 complex. Supported by bioinformatics modeling and *in vitro* results, this surprising finding appears to be related to structural homology between apoA-1 and TLR-2 [30], supporting the molecular mimicry hypothesis to account for this cross-reactivity. Those findings are in accordance with previous reports supporting the general concept that autoantibodies, such as antiphospholipid and anti-HSP antibodies, can promote sterile inflammation through their interaction with different TLRs [46–48].

Another potential proinflammatory mechanism inferred to anti-apoA-1 IgG antibodies is their ability to promote neutrophil chemotaxis toward IL-8 and TNF-*α* (both expressed within atherosclerotic plaques) when compared with CTL medium or CTL IgG treatments, a phenomenon not observed for monocytes [35]. The exact mechanisms accounting for this phenomenon are currently unknown.

Secondly, mirroring the clinical finding showing a positive association between anti-apoA-1 IgG levels and resting heart rate [26], a well-established cardiovascular prognostic feature after MI [27–29], *in vitro* studies demonstrated that in presence of aldosterone, anti-apoA-1 IgG can elicit a dose-dependent increase of the spontaneous contraction rate of neonatal rat ventricular cardiomyocytes [26, 49]. Although still fragmentary, this positive chronotropic effect observed *in vitro* is mediated by L-type calcium channel activation induced by the nongenomic downstream activation of mineralocorticoid receptor, involving phosphatidyl 3-kinase pathways [49]. Interestingly, this chronotropic effect was abrogated by eplerenone but not with aldactone, as well as by intravenous immunoglobulins [26, 49]. Nevertheless, the anti-apoA-1 IgG specific receptor on cardiomyocytes has not been described yet and is under active investigation in our laboratory.

Animal studies appear to provide convergent results with the aforementioned findings. Indeed, Montecucco and colleagues demonstrated that passive immunization of apoE −/− mice under normal chow diet (a validated model of atherosclerosis) increased the size of atherosclerotic lesions and induced a vulnerable phenotype, mirroring the one retrieved in humans consisting in higher intraplaque MMP-9 expression, higher neutrophil content, and lower collagen content when compared to control IgG [35]. In a lupus-prone mice model, the presence of anti-apoA-1 antibodies has been shown to be associated with a decrease in the antioxidant properties of HDL related to a decrease in PON-1 activity leading to an increase of proinflammatory reactive oxygen species [50], corroborating the clinical observations reported earlier in humans [13–15, 17, 18]. 

## 5. Conclusions and Future Perspectives

To summarize, recent studies demonstrate that IgG autoantibodies against apolipoprotein A-1 (apoA-1) are raised in many diseases associated with a high cardiovascular risk, such as SLE, ACS, RA, severe carotid stenosis, and end-stage renal disease. So far, high levels of anti-apoA-1 IgG were shown to be an independent prognostic marker of poor CV outcome in MI and in RA patients, to display clinically relevant properties for NSTEMI diagnosis in acute chest pain patients and to be associated with atherosclerotic plaque vulnerability in patients with severe carotid stenosis. In many studies, high levels of anti-apoA-1 IgG are associated with a proinflammatory cytokine profile, and in SLE/APS, those autoantibodies have been shown to be associated with the presence of dysfunctional HDLs. 

Concomitantly, *in vitro* data tend to indicate that anti-apoA-1 IgG are active modulators of atherogenesis by (i) promoting sterile inflammation through TLR2/CD14 complex and by (ii) eliciting specific neutrophil chemotaxis. Furthermore, intriguing *in vitro* experiments suggests that those autoantibodies could act as a proarythmogenic molecule through an aldosterone-dependent L-type calcium channels activation that can be reverted by existing therapeutic compounds. In parallel, mice models demonstrate that passive immunization with anti-apoA-1 IgG increases atherogenesis, as well as atherosclerotic plaque vulnerability, and decreases the antioxidant properties of HDL by inhibiting PON-1 activity. Taken together, those preliminary results need to be replicated in larger multicentre cohorts, and a better understanding of their physiopathological involvement in atherogenesis is required. Nevertheless, the current converging *in vitro* and animal observations lend weight to the hypothesis that anti-apoA-1 IgG are active mediators of atherogenesis rather than an innocent bystander. If true, those autoantibodies could in the future represent a new possible therapeutic target.

Currently, approximately 20% of patients with patent CVD do not display any Framingham risk factors [51], baring out the importance of identifying new and reversible emergent CVD risk factors. Because antibody-mediated diseases and some cardiovascular conditions can be treated by specific immunologic therapeutic strategies, such as passive immunization [52], anti-apoA-1 IgG could represent an innovative theranostic candidate allowing the identification of subset of CVD that could benefit from this kind of therapy in the future and substantially contribute to personalized medicine in the field of CVD.
